# Effectiveness and adverse events of topical and allergen immunotherapy for atopic dermatitis: a systematic review and network meta-analysis protocol

**DOI:** 10.1186/s13643-020-01472-w

**Published:** 2020-09-28

**Authors:** Luis Guillermo Gómez-Escobar, Hansel Mora-Ochoa, Andrea Vargas Villanueva, Loukia Spineli, Gloria Sanclemente, Rachel Couban, Elizabeth García, Edgardo Chapman, Juan José Yepes-Nuñez

**Affiliations:** 1 School of Medicine, Universidad de los Andes, Bogotá D.C., Colombia; 2 Midwifery Research and Education Unit, Hannover Medical School, Hannover, Germany; 3 Dermatology Section, University of Antioquia, Medellín, Colombia; 4 Department of Health Research Methods, McMaster University, Hamilton, Ontario Canada; 5 Pulmonology Service, Internal Medicine Section, Fundación Santa Fe de Bogotá University Hospital, Bogotá D.C., Colombia

**Keywords:** Atopic dermatitis, Topical administration, Immunotherapy, Systematic review, Meta-analysis, Network meta-analysis, GRADE approach

## Abstract

**Background:**

Atopic dermatitis (AD) is an inflammatory chronic condition that affects the skin of children and adults and has an important impact on the quality of life. Treatments for AD are based on environmental controls, topical and systemic therapies, and allergen-specific immunotherapy (AIT). However, it remains unclear the effectiveness and adverse events of AIT and all conventional topical treatments compared with placebo and each other for AD.

**Methods:**

We will search five electronic databases [Central Cochrane register of controlled trials (CENTRAL), MEDLINE, EMBASE, CINAHL, and LILACS] from inception until November 2019 with no language restriction, and we will include experimental studies [randomized controlled trials (RCTs), and quasi-RCTs]. The primary outcome is global and specific skin symptoms assessment. Secondary outcomes are hospital length of stay, quality of life, and adverse events. Reviewers independently will extract data from the studies that meet our inclusion criteria and will assess the risk of bias of individual primary studies. We will conduct random effects pairwise meta-analyses for the observed pairwise comparisons with at least two trials. Then, we will perform random-effects Bayesian network meta-analysis (NMA) to obtain treatment effects for all possible comparisons and to provide a hierarchy of all interventions for each outcome. Possible incoherence between direct and indirect sources of evidence will be investigated locally (if possible) and globally. To investigate sources of statistical heterogeneity, we will perform a series of meta-regression analyses based on pre-specified important effect modifiers. Two authors will appraise the certainty of the evidence for each outcome applying the GRADE’s framework for NMA.

**Discussion:**

The findings of this systematic review will shed the light on the effectiveness and adverse events of all possible comparisons for treating AD and on the quality of the collated evidence for recommendations. It will also provide critical information to health care professionals to comprehend and manage this disease at different age stages, treatment type, duration, and severity of atopic dermatitis.

**Systematic review registration:**

PROSPERO Protocol ID CRD42019147106

## Background

Atopic dermatitis (AD) is an inflammatory chronic condition of the skin that affects 15 to 20% of children and 3 to 5% of adults worldwide [1–3]. AD prevalence currently seems to be increasing in Africa, East Asia, and some parts of Europe [4]. Its increasing prevalence leads to direct US national costs of $3.8 billion dollars per year [5]. The main clinical features of AD are dry skin, eczema, and pruritus [2]. Pruritic erythematous papule-vesicles characterize acute eczema [6, 7]. Subacute and chronic lesions usually correspond to drier and desquamative lesions with excoriations, lichenification, and fissures areas due to chronic skin scraping [2, 6]. AD has an important impact on the quality of life, as it affects emotional health and socialization, especially when lesions are visible, and symptoms are not controlled [8, 9].

Therapy for AD focus on symptoms improvement, avoidance of exacerbations, and minimizing therapeutic risk [10]. Treatments are based on environmental measures, topical and systemic treatments, and allergen-specific immunotherapy (AIT) [6, 11–15]. Conventional topical treatments such as skin moisturizers and emollients are the first-line treatments due to their effect in maintaining skin hydration [16]. Corticosteroids, calcineurin inhibitors, janus-kinase (JAK) inhibitors, and phosphodiesterase-4 (PDE-4) inhibitors in their topical presentations are recommended in cases of non-controlled AD [6, 12–15, 17]. Further, in severe forms of AD with treatment failure with topical treatment, it is necessary to start a systemic therapy [6]. However, despite all these available treatments, a subgroup of patients remains clinically uncontrolled [10]. These patients have poor quality of life and they are frequently seeking medical attention without achieving adequate control of their symptoms [18].

AIT, an intervention that involves the administration of increasing amounts of a specific allergen to an allergic patient, has demonstrated promising results to decrease the severity of the disease in patients with AD that do not require systemic treatment [19, 20]. Since published evidence shows that AIT modifies the immune response to aeroallergens in patients with allergic rhinitis and asthma, AIT might modify the immune response in patients with AD improving their cutaneous symptoms [21]. However, the efficacy and adverse events of AIT for AD have not been directly or indirectly compared with all conventional topical treatments. Therefore, we aim to compare the effectiveness and adverse events of AIT versus conventional topical treatments or placebo in patients with AD through a systematic review with NMA.

## Methods

This protocol for a systematic review with NMA is registered in PROSPERO (CRD42019147106), an international database of prospectively registered systematic reviews in health, and it will be conducted in accordance to the Cochrane Handbook [22]. For the development of the present protocol, we used to the Preferred Reporting Items for Systematic review and Meta-Analysis Protocols (PRISMA-P) [23] (see Additional file 1).

### Data source and search strategy

We will search MEDLINE, EMBASE, CINAHL, LILACS, and the Cochrane Controlled Register of Trials (CENTRAL) databases for relevant literature from inception until November 2019. A librarian from the Department of Health Research Methods, Evidence, and Impact (HEI) from McMaster University with expertise in designing search strategies for systematic reviews created our search strategy (see Additional file 2). Search will not be restricted to any language, stage, or date of publication. We will also conduct a manual search of RCTs and grey literature through clinicaltrials.gov and the WHO international registry of clinical trials, and summary of conferences, and dissertation databases through ProQuest Dissertations and thesis database. We will contact the authors of non-published works to guarantee eligibility.

### Eligibility criteria

#### Population

Adults or children with mild, moderate, or severe AD, as defined by the investigators, with or without allergic sensitization to an inhalant or food. All countries and settings are eligible for inclusion.

#### Interventions and comparisons

Topical corticosteroids, topical calcineurin inhibitors, topical PDE-4 inhibitors, topical JAK inhibitors, coal tar, topical aryl hydrocarbon receptor activators, subcutaneous AIT or sublingual AIT for any type of allergen, placebo, or standard care. We define standard care as it is defined by the investigators, including skin hydration and moisturization, and bathing with soaps and washes, among others. In the case of co-interventions, we will explore whether the intervention effect is modified by the addition of supplementary intervention, such as the presence of standard care, through subgroup analysis. Comparisons may include individual and/or combined interventions at any dose or presentation.

#### Outcomes

The *primary outcomes* include (1) The proportion of patients (or parents) that inform or present a global improvement of cutaneous symptoms at the end of treatment, and (2) the proportion of patients (or parents) that inform or present an improvement of specific symptoms such as erythema, vesicles, xerosis, excoriation, and/or lichenification of the skin at the end of treatment. *Secondary outcomes* include (3) preventing the development of asthma and/or other allergy diseases such allergic rhinitis and food allergy, (4) the severity of the disease at the end of treatment defined by scores (SCORAD, EASI, or any other used in the study) that assess lesion intensity and/or extension, symptoms, disease course, and epidermal function, (5) changes in the quality of life in both mental and physical health, and (6) local or systemic adverse events.

We will investigate these outcomes by short term (≤ 16 weeks of treatment), and long-term, (> 16 weeks of treatment. We will use data at the end of the study in case of multiple times measurements. Multiple times will also be classified as short term and long term based on data at the end of the study.

#### Study designs

Experimental studies [randomized clinical trials (RCTs) and quasi-RCTs]. We will not limit study inclusion by publication status, the language of dissemination, duration of follow-up, or period of study conduct.

### Study selection

Prior to article selection we will conduct a calibration process to determine the agreement between the reviewers assessing the kappa statistic expecting to get a score greater than 0.7 [22]. Reviewers will go through the search hits by reading titles and abstracts and evaluate its eligibility in an independent and duplicate approach. For each potentially relevant study, we will obtain the full text and will assess its inclusion. In case of disagreement between reviewers, a third author will review the study and resolve its inclusion. Literature search results will be uploaded to Covidence® Software, an Internet-based software program that facilitates collaboration among reviewers during the study selection process.

### Data extraction

We will extract the data, in duplicate, from the eligible studies using a pre-specified Microsoft Excel® form. The following data will be extracted: characteristics of the study (design, year, follow-up duration, sample size per arm, environment, and country), patient characteristics (average age, in- or out-patient setting, duration of the disease since first diagnosis, allergenic sensitization), and intervention characteristics (doses, routes of administration). For binary outcomes, we will extract the number of events, number of missing participant outcome data, and number of randomized participants for each arm of every trial, whereas for continuous outcomes, we will extract the mean, standard deviation, number of missing participant outcome data and number of randomized participants for each arm of every trial. To tackle well-known challenges with the data extraction for continuous outcomes, we will consider the directions described in Section 7.7.3 of the Cochrane Handbook [22]; for instance, to obtain missing standard deviations from reported standard errors, confidence intervals, *t* values, or *p* values for the difference in means. All reviewers will assess the data extraction checklist before the extraction. Five reviewers (LG, AV, HM, AG, and JY) will extract data independently and by duplicate. Any disagreement will be assessed by a third reviewer (EG or JY).

### Assessment of risk of bias in included studies

We will use a modify version of the Cochrane’s Risk of Bias (RoB) tool to assess the RoB in the included studies [24]. In duplicate and independently, we will assess the following domains: sequence for random allocation, allocation concealment, blinding of participants and personnel, blinding of outcome assessment, incomplete outcome data, selective reporting, and other biases. Each domain will be assessed as “definitively yes,” “probably yes,” “probably no,” or “definitively no.” Any disagreement will be assessed by a third reviewer (EC or JY).

### Random-effects pairwise meta-analysis

Clinical and methodological heterogeneity is expected between studies (issue addressed in the section: certainty evaluation in network metanalysis effect estimation) which may lead to statistical heterogeneity. To encompass statistical heterogeneity, random-effects (RE) meta-analysis will be performed for each direct comparison that is informed by at least two trials. Compared to fixed-effect model, RE model [25] inherently leads to wider confidence/credible intervals around the pooled treatment effect depending on the extent of between-trial variance as measured by the parameter *τ*^2^. Under the RE model, the observed treatment effect in a study is a function of the average treatment effect from all studies, the random-effect and the sampling error of that study [26]. In other words, RE model assumes that the true treatment effects of the included trials are randomly sampled from a specific distribution (commonly a normal distribution). Binary outcomes will be analyzed using the odds ratio (OR), whereas continuous outcomes using the mean difference if all trials have used the same scale or standardized mean difference (SMD) when scales are different. To avoid replacing zero cells with abstract thresholds to estimate OR and its variance, we will apply Bayesian RE meta-analysis with binary likelihood and logit link to model the binary outcome data [27]. For continuous outcomes, we will apply Bayesian RE meta-analysis with normal likelihood and identity link [27]. Results will be reported using the posterior mean for the treatment effects but the posterior median for *τ*^2^ alongside their 95% credible intervals. To account for possible missing participant outcome data in the included trials, we will apply the pattern-mixture model with informative missingness odds ratio parameter as proposed by Turner et al. [28] for binary outcomes, and the pattern-mixture model with informative missingness difference of means parameter as proposed by Mavridis et al. [29] for continuous outcomes. Both missingness parameters will be modeled under the missing at random assumption with an independent, uncorrelated structure for each arm of every trial.

### Random-effects network meta-analysis

We expect that many of the available treatments to treat AD have not been compared in any trial. In the absence of direct evidence for a comparison, indirect evidence can be obtained by combining trials that compare the interventions with a common comparator. By incorporating direct and indirect evidence in a single analysis, NMA provides an internally coherent set of relative treatment effects for all possible comparisons and, therefore, allows for a formal hierarchy of the interventions from the best to worst for each outcome [30]. If transitivity assumption (i.e., similarity of included trials in terms of clinical and methodological characteristics that comprise important effect-modifiers) is deemed plausible, NMA can be safely applied to provide credible results [31]. Otherwise, violation of transitivity assumption may cause incoherence between direct and one or more indirect effects beyond between-trial variance, and thus, reduce our certainty to the NMA results [31]. We will assess the plausibility of transitivity assumption by investigating the distribution of important effect modifiers in each observed comparison [32]. We will apply Bayesian RE NMA with consistency equation and incorporation of multi-arms trials to accommodate the anticipated statistically heterogeneity and to account for the correlation between treatment effects that share the same control arm in multi-arm trials [27, 33]. Between-trial variance will be assumed common in the whole network to enable estimation of the parameter for comparisons with few trials, as information is “borrowed” by comparisons with many trials [31]. Under this assumption, the correlation between treatment effects in multi-arm trials equals 0.5 [27]. Coherence, the statistical manifestation of transitivity, will be investigated locally and globally. For the former, we will apply the node-splitting approach [34, 35] using the R-package gemtc [36] to automatically identify the comparisons to split in closed loops of interventions, whereas for the latter, we will compare the model-fit and complexity of the NMA model with and without consistency equation using the deviance information criterion (DIC) which provides a measure of model fit penalized for model complexity [37]. The model with lower DIC will be considered to have a better compromise between model fit and complexity [34, 38].

To illustrate the network geometry, we will create network plots for each outcome. The plots will display visual information of the evidence retrieved for each outcome regarding the number of trials and patients involved in each direct comparison.

In line with the statistical analysis for pairwise meta-analysis, we will use OR and mean difference or SMD as effect measures for binary and continuous outcomes respectively, and we will report the posterior mean for the treatment effects but the posterior median for *τ*^2^ alongside their 95% credible intervals. Furthermore, we will extent the pattern-mixture model of Turner et al. [28] for binary missing participant outcome data to operate in a network of interventions [39], and we use the pattern-mixture model of Mavridis et al. [29] to incorporate continuous missing participant outcome data as observed in the analyzed networks. We will create league tables for each outcome to present the NMA results for all possible comparisons as well as the results from pairwise meta-analysis for the observed comparisons with at least two trials. Furthermore, for each outcome, we will create forest-plots to present the NMA treatment effects alongside the respective direct and indirect treatment effects of comparisons with the reference intervention of the network. We will also present several measures of intervention hierarchy including the rank probabilities, ranks, and surface under the cumulative ranking curve (SUCRA) values [40]. Specifically, we will create rankograms for each intervention and outcome to fully illustrate the uncertainty across the ranks [40]. We will also present SUCRA plots to illustrate the cumulative ranking probabilities for each intervention and outcome [40]; for each intervention, SUCRA value indicates the percentage of effectiveness (or safety) of that intervention as compared to an imaginary intervention that is always the best with certainty [41]. For each outcome, the best treatment will have high SUCRA value and the worst treatment low SUCRA value. To aid the interpretation of the results in terms of hierarchy and relative treatment effects, we will incorporate the posterior median ranks and posterior mean SUCRA values alongside their 95% credible interval in the aforementioned forest-plots. We will create a scatter diagram to identify the best balance between efficacy and safety.

### Investigating statistical heterogeneity

Thirteen a priori important effect-modifiers will be considered to investigate possible incoherence and statistical heterogeneity. Possible effect-modifiers are (1) severity of the disease: mild/moderate vs severe (based on SCORAD scale); (2) allergy sensitization proven by positive skin prick test or circulating levels of allergen-specific IgE antibody or total IgE detected vs no allergy sensitization proven; (3) pediatric vs adult population (18 years or older); (4) route of allergenic immunotherapy administration: sublingual vs subcutaneous; (5) duration of treatment: short term (less than 16 weeks) vs long term (more than 16 weeks); (6) type of allergen: dust mites vs pollen vs pet allergen; (7) adverse events: local vs systemic; (8) small-study effects; (9) RoB level of the trials included: low vs high risk of bias; (10) funding resource (pharmaceutical companies, grants or other financial resource); (11) treatment type: topical corticosteroids, calcineurin inhibitors, topical PDE-4 inhibitors, JAK topical inhibitors, sublingual allergen immunotherapy, subdermal allergen immunotherapy; (12) same standard care intervention is delivered to both the intervention and comparator groups; and (13) presence of other allergic diseases such as asthma, allergic rhinitis, and food allergy. We anticipate a stronger treatment effect in patients with severe AD, subcutaneous AIT, patients with allergy sensitization proved, duration of more than 16 weeks, in smaller trials, and patients with other allergic diseases. In case of bias, RCTs with a higher RoB may show bigger treatment effects than RCTs with lower RoB. The impact of the effect-modifiers on the NMA results will be investigated by applying Bayesian meta-regression RE models and assuming exchangeable regression coefficients [42].

### Assessment of small-study effects and possible publication bias

Since different studies investigate different comparisons, we will provide a comparison-adjusted funnel plot to investigate graphically the presence of possible small-study effects [22, 43]. This plot is an extension of the funnel plot used in pairwise meta-analysis, as it distinguishes among the different trial-specific treatment effects for different comparisons [43]. We will apply three comparison-adjusted funnel plots where studies will be labeled as (i) active- versus placebo-controlled trials, (ii) old versus new intervention, and (iii) sponsored versus non-sponsored intervention. In the presence of funnel plot asymmetry, we will use selection model to investigate the possibility of publication bias [44].

### Model specification in pairwise and network meta-analysis

In both pairwise and network meta-analysis, prior normal distributions centered at 0 with variance equal 10,000 will be used for all location parameters of the models, whereas for the parameter *τ*^2^ we will use proper empirical priors tailored to the intervention-comparison type and the investigated outcome as suggested by Turner el al [45]. for binary outcomes and Rhodes et al. [46] for continuous outcomes. For the meta-regression analyses, prior normal distribution centered on 0 with variance equal 10,000 and uniform distribution over the interval [0, 5] will be assigned on the common mean and standard deviation of the normally distributed regression coefficients, respectively. For all Bayesian analyses, we will apply three parallel chains with different initial values using 200,000 updates and a burn-in of 20,000 MCMC samples. We will assess convergence using the Gelman–Rubin convergence diagnostic, $\hat{R}$, and through inspection of trace and autocorrelation plots [47]. All analyses will be performed in JAGS [48] using the R-package gemtcs [36] and R2jags [49].

### Classifying certainty of effect estimates in NMA

The reviewers will evaluate in pairs and independently the certainty of estimations (quality of evidence) for each informed outcome according to GRADE [50]. Certainty will be classified in four levels: high, moderate, low, and very low. We will evaluate and classify each direct comparison result according to the following categories: RoB [51], inconsistency (determined by heterogeneity as previously mentioned) [52], indirectness [53], and publication bias [54]. We will also assess imprecision on the credible intervals around the network estimates according to the GRADE for NMA updated guidance [27].

To evaluate the certainty of the NMA estimations, we will follow four steps: (1) present the direct and indirect estimates of effect for the pairwise comparison, (2) rate the certainty of both of these estimates, (3) present the network estimate for the pairwise comparison, and (4) rate the certainty of the network estimate, based on the ratings of the direct and indirect estimates and the assessment of coherence. For rating the certainty of the indirect estimates, we will focus their assessment on the most-dominant first-order loop [26]. We will assess the certainty of indirect effect estimates if the certainty of the direct estimates is not high, and the contribution of the direct evidence to the network estimate is lower as that of the indirect evidence [27].

Our judgment of certainty in the NMA estimation to any paired comparison will be the highest of the certainty qualifications between the direct and indirect comparisons that contribute to the model. Nonetheless, we can reduce the certainty in the network estimation if we find that direct and indirect estimations are inconsistent and/or imprecise. Using the updated GRADE approach we will also assess incoherence or inconsistency, which is defined as the effect difference between direct and indirect estimations [55].

## Discussion

To our knowledge, this protocol describes the first systematic review and network meta-analyses that specifically examines the effectiveness and adverse events of topical and allergen immunotherapy for atopic dermatitis. Previous reviews have described the efficacy of allergen-specific immunotherapy for atopic dermatitis [56], the efficacy of subcutaneous and sublingual grass allergen immunotherapy [57], and a Cochrane review about specific allergen immunotherapy for the management of atopic dermatitis [58]. This systematic review will describe the proportion of patients with global improvement of cutaneous symptoms, proportion of patients that present improvement in specific symptoms, the severity of the disease at the end of treatment, changes in quality of life, and adverse events across multiple interventions. Our NMA will allow the comparison and ranking of treatments that have not been compared head to head.

Our target users are allergy and dermatology practitioners, as well as researchers and healthcare policy-makers. We plan to present our results at national and international meetings.

Limitations to the review may include the diverse outcome measurements and differences in trial design that may limit our capacity to combine results from different clinical trials. Additionally, limited data in certain interventions may limit the ability to run an NMA on all outcomes described.

The findings from this systematic review and NMA will help health care professionals to make evidence-based decisions for AD patients in the absence of head to head trial comparisons in topical and AIT interventions. Our findings will also identify evidence gaps and decrease uncertainty in relative and absolute estimates of the interventions being compared.

## Supplementary information

**Additional file 1.** PRISMA-P 2015 Checklist.

**Additional file 2.** Medline Search Strategy.

## Data Availability

The datasets created and analyzed during this review will be available from the corresponding author upon reasonable request.

## Abbreviations

AD: Atopic dermatitis
AIT: Allergen-specific immunotherapy
GRADE: Grading of Recommendation, Assessment, Development and Evaluation
JAK: Janus kinase
NMA: Network meta-analysis
OR: Odds ratio
PDE-4: Phosphodiesterase-4
RCT: Randomized clinical trials
RoB: Risk of bias
SMD: Standard mean difference
SUCRA: Surface under the cumulative ranking curve
