# Supplementary material for: Effectiveness and adverse events of topical and allergen immunotherapy for atopic dermatitis: a systematic review and network meta-analysis protocol
Source: Syst Rev. 2020 Sep 28;9:222. doi: 10.1186/s13643-020-01472-w (PMC7523328; doi:10.1186/s13643-020-01472-w)
Supplement: Supplementary file 2 — Additional file 2. Medline Search Strategy. [file 13643_2020_1472_MOESM2_ESM.docx]

**Additional File 2. Medline Search Strategy**

|  |  | **MEDLINE (Ovid)** |
| --- | --- | --- |
| Design | 1 | Randomized Controlled Trials as Topic/ |
|  | 2 | randomized controlled trial/ |
|  | 3 | Random Allocation/ |
|  | 4 | Double Blind Method/ |
|  | 5 | Single Blind Method/ |
|  | 6 | clinical trial/ |
|  | 7 | clinical trial, phase i.pt |
|  | 8 | clinical trial, phase ii.pt |
|  | 9 | clinical trial, phase iii.pt |
|  | 10 | clinical trial, phase iv.pt |
|  | 11 | controlled clinical trial.pt |
|  | 12 | randomized controlled trial.pt |
|  | 13 | multicenter study.pt |
|  | 14 | clinical trial.pt |
|  | 15 | exp Clinical Trials as topic/ |
|  | 16 | (clinical adj trial$).tw |
|  | 17 | ((singl$ or doubl$ or treb$ or tripl$) adj (blind$3 or mask$3)).tw |
|  | 18 | PLACEBOS/ |
|  | 19 | placebo$.tw |
|  | 20 | randomly allocated.tw |
|  | 21 | (allocated adj2 random$).tw |
|  | 22 | OR/1-21 |
|  | 23 | case report.tw |
|  | 24 | letter/ |
|  | 25 | historical article/ |
|  | 26 | OR/23-26 |
|  | 27 | 22 NOT 26 |
| Participants | 28 | exp Eczema/ |
|  | 29 | exp Dermatitis, Atopic/ |
|  | 30 | exp Dermatitis/ |
|  | 31 | eczema.mp. |
|  | 32 | atopic eczema.mp. |
|  | 33 | atopic dermatitis.mp. |
|  | 34 | neurodermatitis.mp. |
|  | 35 | besnier$.mp. |
|  | 36 | prurigo.mp. |
|  | 37 | OR/28-36 |
| Int. AIT | 38 | exp Desensitization, Immunologic/ or desensitization.mp. |
|  | 39 | immunotherapy.mp. or exp Immunotherapy/ |
|  | 40 | immunomodulatory.mp. |
|  | 41 | immune therapy.mp. |
|  | 42 | hyposensitisation.mp. |
|  | 43 | specific allergen immunotherapy.mp. |
|  | 44 | SLIT.mp. |
|  | 45 | SCIT.mp. |
|  | 46 | (topical$ adj3 corticosteroid$).ti,ab. |
|  | 47 | (topical$ adj3 steroid$).ti,ab. |
| Int. Glucocortidoids | 48 | exp Desonide/ |
|  | 49 | alclometasone dipropionate.mp. |
|  | 50 | amcinonide.mp. |
|  | 51 | Beclomethasone/ |
|  | 52 | beclometasone dipropionate.mp. |
|  | 53 | exp Betamethasone/ |
|  | 54 | betamethasone benzoate.mp. |
|  | 55 | Betamethasone butyrate propionate.mp. |
|  | 56 | betamethasone dipropionate.mp. |
|  | 57 | (betamethasone adj2 valerate).mp. |
|  | 58 | exp Budesonide/ |
|  | 59 | budesonide.mp. |
|  | 60 | exp Clobetasol/ |
|  | 61 | clobetasol.mp. |
|  | 62 | clobetasone.mp. |
|  | 63 | clocortolone pivalate.mp. |
|  | 64 | (exp Cortisone/ or cortisone.ti,ab.) and (exp Administration, Topical/) |
|  | 65 | Deprodone propionate.mp. |
|  | 66 | desonide.mp. |
|  | 67 | exp Desonide/ |
|  | 68 | exp Desoximetasone/ |
|  | 69 | desoximetasone.mp. |
|  | 70 | exp Dexamethasone/ |
|  | 71 | dexamethasone.mp. |
|  | 72 | diflorasone.mp. |
|  | 73 | exp Diflucortolone/ |
|  | 74 | diflucortolone.mp. |
|  | 75 | fluclorolone.mp. |
|  | 76 | fludroxycortide.mp. |
|  | 77 | flumetasone.mp. |
|  | 78 | exp Flumethasone/ |
|  | 79 | exp Fluocinolone Acetonide/ |
|  | 80 | fluocinolone acetonide.mp. |
|  | 81 | exp Fluocinonide/ |
|  | 82 | fluocinonide.mp. |
|  | 83 | fluocortin.mp. |
|  | 84 | exp Fluocortolone/ |
|  | 85 | fluocortolone.mp. |
|  | 86 | fluprednidene.mp. |
|  | 87 | flurandrenolide.mp. |
|  | 88 | exp Flurandrenolone/ |
|  | 89 | flurandrenolone acetonide.mp. |
|  | 90 | fludroxycortide.mp. |
|  | 91 | fluticasone.mp. |
|  | 92 | exp Halcinonide/ |
|  | 93 | halcinonide.mp. |
|  | 94 | halobetasol.mp. |
|  | 95 | halometasone.mp. |
|  | 96 | exp Hydrocortisone/ |
|  | 97 | (hydrocortisone or cortisol).ti,ab. and (exp Administration, Topical/) |
|  | 98 | (hydrocortisone or cortisol).ti,ab. and (exp Ointments/ or Dermatologic Agents/) |
|  | 99 | hydrocortisone butyrate.mp. |
|  | 100 | hydrocortisone aceponate.mp. |
|  | 101 | hydrocortisone acetate.mp. |
|  | 102 | hydrocortisone valerate.mp. |
|  | 103 | masipredone hydrochloride.mp. |
|  | 104 | exp Methylprednisolone/ |
|  | 105 | methylprednisolone aceponate.mp. |
|  | 106 | methylprednisolone acetate.mp. |
|  | 107 | mometasone.mp. |
|  | 108 | prednicarbate.mp. |
|  | 109 | (exp Prednisolone/ or prednisolone.ti,ab.) and (exp Administration, Topical/ ) |
|  | 110 | (exp Prednisolone/ or prednisolone.ti,ab.) and (exp Ointments/ or Dermatologic Agents/) |
|  | 111 | Prednisolone valerate acetate.mp. |
|  | 112 | exp Triamcinolone/ |
|  | 113 | triamcinolone.mp. |
|  | 114 | ulobetasol.mp. |
|  | 115 | exp Adrenal Cortex Hormones/ and (exp Administration, Topical/ or exp Ointments/ or Dermatologic Agents/) |
|  | 116 | exp Glucocorticoids/ and (exp Administration, Topical/ or exp Ointments/ or Dermatologic Agents/) |
|  | 117 | (topical$ adj3 glucocorticoid$).mp. |
|  | 118 | (topical$ adj3 corticoid$).mp. |
| Int. Calcineurin in. | 119 | exp Calcineurin Inhibitors/ |
|  | 120 | calcineurin inhibitors.mp |
|  | 121 | tacrolimus.mp. |
|  | 122 | prograf?.mp |
|  | 123 | FK?506.mp. |
|  | 124 | pimecrolimus.mp. |
|  | 125 | Elidel.mp. |
|  | 126 | ASM?981.mp |
| PDE4 in. | 127 | exp Inhibitors, Phosphodiesterase 4/ |
|  | 128 | Phosphodiesterase 4 Inhibitors.mp. |
|  | 129 | PDE?4 inhibitors.mp. |
|  | 130 | crisaborole.mp. |
|  | 131 | eucrisa.mp. |
|  | 132 | AN-2728.mp. |
| Other | 133 | tapinarof.mp. |
|  | 134 | WBI-1001.mp. |
|  | 135 | benvitimod.mp. |
|  | 136 | exp Tal, Coal/ |
|  | 137 | coal tar.mp. |
| JAK in. | 138 | exp Inhibitors, Janus Kinase/ |
|  | 139 | JAK inhibitors.mp. |
|  | 140 | tofacitinib.mp. |
|  | 141 | tasocitinib.mp. |
|  | 142 | CP?690?550.mp. |
|  | 143 | JTE-052.mp. |
|  | 144 | OR/38-143 |
|  | 145 | 27 AND 37 AND 144 |
